# Precision Medicine Approach to Alzheimer’s Disease: Rationale and Implications

**DOI:** 10.3233/JAD-230467

**Published:** 2023-11-07

**Authors:** Dale E. Bredesen, Kat Toups, Ann Hathaway, Deborah Gordon, Henrianna Chung, Cyrus Raji, Alan Boyd, Benjamin D. Hill, Sharon Hausman-Cohen, Mouna Attarha, Won Jong Chwa, Alexei Kurakin, Michael Jarrett

**Affiliations:** a Department of Molecular and Medical Pharmacology, David Geffen School of Medicine, UCLA, Los Angeles, CA, USA; b Bay Area Wellness, Walnut Creek, CA, USA; c Dr. Ann Hathaway, San Rafael, CA, USA; d Northwest Memory Center, Ashland, OR, USA; e Quesgen Systems, Burlingame, CA, USA; f Department of Radiology, Washington University School of Medicine, St. Louis, MO, USA; g CNS Vital Signs, Morrisville, NC, USA; h Department of Psychology, University of South Alabama, Mobile, AL, USA; i IntellxxDNA, Austin, TX, USA; j Posit Science, San Francisco, CA, USA; k Department of Radiology, St. Louis University, St. Louis, MO, USA; l Department of Neurology, David Geffen School of Medicine, UCLA, Los Angeles, CA, USA

**Keywords:** Alzheimer’s disease, clinical trial, mild cognitive impairment, MRI volumetrics, neurodegeneration, systems

## Abstract

The neurodegenerative disease field has enjoyed extremely limited success in the development of effective therapeutics. One potential reason is the lack of disease models that yield accurate predictions and optimal therapeutic targets. Standard clinical trials have pre-determined a single treatment modality, which may be unrelated to the primary drivers of neurodegeneration. Recent proof-of-concept clinical trials using a precision medicine approach suggest a new model of Alzheimer’s disease (AD) as a chronic innate encephalitis that creates a network insufficiency. Identifying and addressing the multiple potential contributors to cognitive decline for each patient may represent a more effective strategy. Here we review the rationale for a precision medicine approach in prevention and treatment of cognitive decline associated with AD. Results and implications from recent proof-of-concept clinical trials are presented. Randomized controlled trials, with much larger patient numbers, are likely to be significant to establishing precision medicine protocols as a standard of care for prevention and treatment of cognitive decline. Furthermore, combining this approach with the pharmaceutical approach offers the potential for enhanced outcomes. However, incorporating precision medicine approaches into everyday evaluation and care, as well as future clinical trials, would require fundamental changes in trial design, IRB considerations, funding considerations, laboratory evaluation, personalized treatment plans, treatment teams, and ultimately in reimbursement guidelines. Nonetheless, precision medicine approaches to AD, based on a novel model of AD pathophysiology, offer promise that has not been realized to date with monotherapeutic approaches.

## INTRODUCTION

Neurodegenerative diseases such as Alzheimer’s disease (AD), Lewy body disease, frontotemporal dementia, and amyotrophic lateral sclerosis are without therapeutics that effect sustained improvements. There are approximately six million people with AD in the United States, and one study estimates that it has become the third leading cause of death [1]. Unfortunately, the best results from recent clinical trials have been to slow cognitive decline rather than improve cognition or halt decline, and are complicated by side effects such as brain edema and microhemorrhage [2, 3], as well as brain atrophy [4].

The etiology of AD remains controversial, and simple, mono-etiological theories such as the theory that AD is “type 3 diabetes” [5], or is due to chronic *Herpes simplex* infection [6], or due to amyloid-β [7], or to misfolded proteins such as tau [8], or prions [9], have not led to effective treatments. However, epidemiological, pathological, toxicological, genetic, and biochemical studies have provided additional candidate mechanisms for the neurodegeneration associated with AD, such as neuroinflammation [10], insulin resistance [11], and reduction in trophic support [12].

We have previously proposed, based on the dichotomous signaling of amyloid-β protein precursor (AβPP), that AD is the result of a network insufficiency [13], triggered by chronic or repeated mismatches between network support (cerebral blood flow, oxygen saturation and substrate availability, mitochondrial function, and trophic support) and demand (which is increased by inflammation, toxic exposure, and stress). Thus, the loss of synapses in AD is viewed as the result of synaptoclastic signaling that is not matched by synaptoblastic signaling, to use an osteoporosis analogy. In this conceptualization of AD, amyloid-β oligomer production is part of a physiological response involving the innate immune system [14], and given the long half-life, amyloid-β is well suited to play a role in trained immunity. AD is thus the result of a chronic, low-grade innate encephalitis that may result from many contributions— central or peripheral, acting independently or synergistically— that activate innate immunity, drive neuroinflammation, and/or lead to energetic reduction. Therefore, individuals who exhibit hyper-responsiveness to various pathogens or inflammagens— such as individuals with the ɛ4 allele of apolipoprotein E, various pro-inflammatory single-nucleotide polymorphisms, or epigenetic alterations (e.g., trained innate immunity)— are at increased risk for this syndrome, with energetic reductions contributing to reduced adaptive responses and pathogen clearing.

Therefore, identifying for each person the contributors to reduced support and/or increased demand of cerebral synaptic networks represents a means to crafting a rational therapeutic approach, and personalized, precision medicine protocols are designed to achieve this goal. Deployment of such a protocol has led to anecdotal reports of cognitive improvement in patients with AD at the stage of mild cognitive impairment (MCI) as well as dementia [15–17], and more recently successful proof-of-concept trials (i.e., leading to improved cognition, not simply slowing decline) [13]. A somewhat similar approach has been reported by Roach and colleagues [18], as well as by Isaacson and colleagues [19].

In the following sections, we outline the methods used in the evaluation, treatment, and outcomes assessment of patients with cognitive decline who are treated with a precision medicine protocol.

## THERAPEUTIC APPROACH: EVALUATION

The goal of the evaluation is to identify the contributors to the proposed network insufficiency, with the four major groups of contributors being: 1) pro-inflammatory agents and signals; 2) toxins and toxicants (inorganics, organics, and biotoxins); 3) energetics (cerebral blood flow, oxygen saturation, mitochondrial function, and substrate concentration); and 4) trophic support (neurotrophic factors, hormones, and nutrients).

Therefore, laboratory evaluation includes markers of inflammation (high-sensitivity C-reactive protein, albumin:globulin ratio, fibrinogen, tumor necrosis factor alpha, omega-6:omega-3 ratio, homocysteine, and uric acid), autoimmune markers (such as CD57, anti-thyroglobulin, anti-thyroid peroxidase, and anti-nuclear antigen), immune markers such as immunoglobulins and lymphocyte subsets, as well as potential sources of chronic or recurring inflammation: chronic pathogens such as *Herpes* family viruses, tick-borne pathogens, *SARS-CoV-2*, *Toxoplasma gondii*, or *Chlamydia pneumoniae*; intestinal hyperpermeability markers (such as antibodies to zonulin or lipopolysaccharide), oral pathogens (such as *P. gingivalis, T. denticola, P. intermedia,* and *F. nucleatum*).

Energetic support evaluation includes measurement of nocturnal SpO_2_ to screen for sleep apnea and upper airway resistance syndrome, identification of insulin resistance (HOMA-IR, hemoglobin A1c), lipid panel, mitochondrial function (organic acid tests), markers of hypercoagulation (such as Factor V Leiden and prothrombin mutations, anti-phospholipid antibodies, lipoprotein (a), protein C and protein S activity), and advanced lipid panels.

Toxin and toxicant evaluation includes screening for exposure to metals (organic and inorganic mercury, lead, cadmium, copper, zinc, iron) and the metalloid arsenic, organics (such as benzene, toluene, glyphosate, and formaldehyde), and biotoxins (such as trichothecenes, ochratoxin A, gliotoxin, and zearalenone). Many of these potential contributors, such as biotoxins, have not been formally recognized as contributing to the cognitive decline associated with AD, but their ability to increase the burden of inflammation and reduce energetic support for the brain makes them strong candidates, and anecdotal evidence implicates them [20].

Trophic support evaluation includes neurotrophins (brain-derived neurotrophic factor (BDNF)), nutrients (B vitamins, vitamin D, vitamin E, magnesium, zinc, copper, CoQ10, lipoic acid, omega-6:omega-3 ratio, omega-3 index), and hormones (estradiol, progesterone, testosterone, pregnenolone, sex-hormone binding globulin, DHEA sulfate, and thyroid).

Genetic testing is carried out, focused especially on genetic variants related to inflammation, methylation, detoxification, hypercoagulability, neurotrophins, neurotransmitters, mitochondrial function, nutrient metabolism, hormone metabolism and signaling, antioxidation, and metal binding.

Imaging is carried out utilizing brain MRI with regional volumetrics.

For some patients, electrophysiological tests are also included, with special attention paid to the P300 evoked response (P300a and b), dominant alpha rhythm on EEG, and theta:beta ratio on EEG.

Valuable new tests, unavailable at the time of the previous trial but incorporated into the ongoing randomized controlled trial (https://www.dementiareversaltrial.com/; https://beta.clinicaltrials.gov/study/NCT05894954?cond=Cognitive% 20Decline&term=reversal&rank=5), should improve accuracy in both diagnosis and follow-up. These tests include epigenetic evaluation as well as blood tests for biomarkers such as p-tau 181, p-tau 217, Aβ42 : 40 ratio, GFAP, and neurofilament light.

From these laboratory tests, potential contributors to cognitive decline are identified. It should be clarified that, unlike in previous teaching that AD should be distinguished from “treatable causes of dementia” (such as normal pressure hydrocephalus or neurosyphilis), here these various treatable factors are potential contributors to AD pathophysiology, not unrelated comorbidities. AD is thus treated as a chronic innate encephalitis that represents a response to these various insults, much as the pathophysiology of multiple sclerosis has turned out to represent a response to *Epstein-Barr* virus (and potentially other viruses) [21], with the key distinction being that multiple sclerosis involves dysregulated adaptive immunity (autoimmunity) [22], whereas AD may primarily involve innate immunity [23].

In addition to determining the potential contributors to AD, the evaluation stratifies patients into AD subtypes in accord with the dominant contributor(s) [24]: inflammatory, glycotoxic, atrophic, toxic, vascular, or traumatic. However, in most patients, multiple subtypes are present.

## THERAPEUTIC APPROACH: TREATMENT

The results of the evaluation of each patient implicate specific contributors and their associated pathways and mechanisms, which are then addressed with a personalized protocol. However, there are core considerations that are included for every patient, such as the goal of achieving insulin sensitivity and metabolic flexibility, i.e., the ability to alternate utilization of glucose and ketones. The defect in glucose utilization in the temporal and parietal brain regions (and especially in the posterior cingulate and precuneus) has been well documented [25], as has the increased risk of cognitive decline in individuals with insulin resistance [26]. Furthermore, the use of exogenous ketones has been shown to improve cognitive function in patients with MCI [27].

Thus, the goal is to identify and address the factors associated theoretically and epidemiologically (though in some cases yet to be proven causally) with AD-related cognitive decline, both common and patient-specific:

• Optimize energetic support (oxygenation, cerebral blood flow, substrate availability, and mitochondrial function);
• Restore insulin sensitivity;
• Improve hyperlipidemia;
• Resolve inflammation if present (and remove the cause(s) of the inflammation);
• Treat identified pathogens;
• Optimize trophic support (hormones, nutrients, and neurotrophic factors);
• Treat autoimmunity if identified;
• Detoxify if toxins are identified.

This approach requires more extensive evaluation than is the current standard of care for patients presenting with cognitive decline, as well as a more complex treatment regimen, and a treatment team that is most effective when including a health coach, nutritionist, and a physical trainer, along with the physician.

The goals of the nutritional component of the protocol are mild ketosis (1.0–4.0 mM beta-hydroxybutyrate) and insulin sensitivity (i.e., metabolic flexibility), microbiome optimization, healing of any gastrointestinal hyperpermeability, avoidance of malabsorption, detoxification, and supply of key nutrients for cognition, such as choline, vitamin B12, and vitamin D. To accomplish these goals, a plant-rich, high-fiber (soluble and insoluble), mildly ketogenic diet, high in leafy greens and other non-starchy vegetables (raw and cooked), high in unsaturated fats (both monounsaturated and polyunsaturated), low in glycemic load, with a fasting period of 12–16 h each night is recommended, and glucose and ketone levels are followed. It is noteworthy that this nutritional approach often leads to improvement in blood pressure, lipid profiles, and glycemic control. Toxicant-minimized produce (often labeled “organic”), wild-caught low-mercury fish (salmon, mackerel, anchovies, sardines, and herring), and modest consumption of pastured eggs and meats are allowed, and avoidance of processed food, simple carbohydrates, grains, and dairy are recommended. Blood ketone levels are monitored with fingerstick ketone meters, with a goal of 1.0–4.0 mM beta-hydroxybutyrate, or less desirably with breathalyzers monitoring acetone levels. The importance of including ketosis as a goal has been supported by the work of Cunnane et al. [28].

The goals of the exercise component of the common part of the protocol are to improve cardiovascular and endothelial functions and insulin sensitivity, enhance ketosis, BDNF, cerebral blood flow, and sleep. Both aerobic and strength training are recommended for at least 45 min per day, at least six days per week (for aerobic exercise) and at least twice per week (for strength training), and this may be facilitated by personal trainers. Balance training is also encouraged. High-intensity interval training (HIIT) is recommended a minimum of twice per week for those capable of performing HIIT.

Sleep hygiene is recommended to ensure 7–8 h of sleep per night, and patients without a diagnosis of sleep apnea are tested over several nights using home sleep study devices. In those diagnosed with sleep apnea or upper airway resistance syndrome (UARS), referral for treatment with a continuous positive airway pressure apparatus or a dental splint device (for those identified with UARS) is provided. Sleep stages are monitored with a wearable device, with a goal of at least one hour of deep, slow-wave sleep per night, and at least 90 min of REM sleep.

Stress is another potential contributor to the cognitive decline associated with AD [29], and therefore management of stress is included as a core component. There are many techniques to address stress as a contributor, with a goal of increasing vagal tone and improving associated heart-rate variability. These include shinrin-yoku [30], transcendental meditation [31], yoga [32], and biofeedback [33], among others.

Although the effect of brain training on cognitive decline has met with some controversy [34], Merzenich and his group have developed and validated brain training approaches to enhance neuroplasticity [35], and therefore brain training is included as a core component of the overall protocol. A HIPAA and SOC-2-compliant platform with empirical validation [36] is utilized, for a minimum of 15 min daily. Participants train on 29 cognitive exercises that target the speed and accuracy of information processing.

For patients in whom suboptimal neurotrophic status is detected (e.g., by serum testing or inference from sedentary lifestyle), BDNF is increased with whole coffee fruit extract (as well as exercise and ketosis) [37]. For those with suboptimal nutrients (e.g., vitamin D, omega-3, B vitamins, CoQ10, or minerals), appropriate nutrients are provided. For those in whom hormone levels are suboptimal, bio-identical hormone replacement and appropriate supplements are provided to optimize sex hormone levels [38], neurosteroids (dehydroepiandrosterone, pregnenolone, and vitamin D), and thyroid medications as indicated for sub-optimal thyroidfunction.

For those found to have gastrointestinal hyperpermeability, infections, inflammation, or impaired absorption and digestion, gut healing with dietary restriction, gut-healing nutrients, and digestive enzyme support if indicated, along with treatment of any identified dysbiosis, is included in the protocol. Gastrointestinal hyperpermeability is assessed by testing for antibody response to permeability-related antigens such as actomyosin, occludin/zonulin, and lipopolysaccharide.

For those with evidence of systemic inflammation, pro-resolving mediators and anti-inflammatory herbs and supplements (such as liposomal glutathione or S-acetyl glutathione, fish oil, resveratrol, vitamins C and D, boswellia, turmeric, and/or quercetin) are included, and low-dose naltrexone is prescribed for those with evidence of autoimmunity. Omega-3 fats are included via diet and supplementation. Note that low-dose naltrexone was chosen for those with autoimmunity because of its ability to increase endorphins, which in turn bind to lymphocyte receptors and regulate immune function, reducing autoimmune responses [39].

As noted above, Itzhaki and other investigators have made a compelling case for a role for microbes in AD pathogenesis [6]. Moreover, as reported by Moir and their colleagues, the oligomeric Aβ peptide is a potent anti-microbial peptide [40], which, similar to other antimicrobial peptides, is produced in response to infections. Therefore, infectious agents associated with cognitive decline or systemic inflammation are identified and treated. For those with evidence of *Herpes simplex* infection or a history of outbreaks, valacyclovir is prescribed for 3–12 months. Active *Epstein-Barr Virus* (EBV) is treated with herbal protocols (such as juniperus, acer, and tamarix or monolaurin, lysine, and olive leaf extract). For those with evidence of tick-borne infections [41] such as *Borrelia*, *Babesia*, or *Bartonella*, organism-sensitive treatment is prescribed with herbal anti-microbials, such as Cryptolepis and Japanese knotweed [42], along with immune support.

For those with toxicity associated with metals (e.g., mercury or lead), organic pollutants (e.g., benzene, phthalates, or organophosphate insecticides), or biotoxins (e.g., trichothecenes, ochratoxin A, or gliotoxin), detoxification is undertaken, starting with identification and avoidance of exposure, and adding binding agents (e.g., cholestyramine, activated charcoal, or bentonite clay), sauna, herbs, sulforaphane, and dietary restriction of seafood if indicated.

## THERAPEUTIC APPROACH: OUTCOMES

Treatment efficacy, and guidance for protocol optimization, are inferred from cognitive tests (Montreal Cognitive Assessment and more sensitive on-line testing with a neurocognitive battery), progress (or lack thereof) in brain training scores, AQ-21-based symptom-change scales as determined by each patient’s partner, and MRI-based brain regional volumetrics. New blood-based biomarker tests such as p-tau 181, p-tau 217, Aβ 42 : 40 ratio, neurofilament light (NfL), and glial fibrillary acidic protein (GFAP), should provide pivotal, complementary, molecular-level data to support or refute the estimation of improvement (cognitive and pathophysiological), and may ultimately offer new insight into the optimal protocols for preventing and reversing cognitive decline.

As the results of the previous trials [13, 43] suggest, multiple health indices are responsive to the protocol described here: metabolic effects observed in the proof-of-concept trial [13] include a significant reduction in serum high-sensitivity C-reactive protein, a significant reduction in glycation (hemoglobin A1c), a reductive trend in insulin resistance (HOMA-IR, homeostasis model assessment-estimated insulin resistance), a significant improvement in lipid profile (reduction in triglyceride-to-high-density-lipoprotein ratio), and a significant increase in serum vitamin D (25-hydroxycholecalciferol) levels.

Significant improvements in cognition were determined by partner-assessed, AQ-21-derived change scale, Montreal Cognitive Assessment scores (which improved in 76% of patients), CNS-Vital Signs on-line assessments (which improved in 84% of patients), and cognitive training scores (which improved in all patients).

Volumetric MRI studies revealed that, over the course of the 9-month study, grey matter volumes increased by an average of 0.3% on an annualized basis, in contrast to the typical decline (documented in previous studies, with a different study population) of 2.20–2.37% for those with AD [44], and 0.83–0.92% reduction per year for those without cognitive decline [45].

Hippocampal volumes of the trial patients decreased at an annualized rate of 1.29%, which is less than the average rate of 3.5% –4.66% in patients with AD, and also less than the average of 1.41–1.73% in cognitively stable controls [46, 47].

It is noteworthy, especially with regard to the serious adverse events that often occur with anti-amyloid antibodies, that no serious adverse events were recorded in this study. On the contrary, most patients improved their overall health, and some patients no longer required anti-hypertensives, anti-diabetes drugs, or lipid-lowering agents.

These metabolic, cognitive, and imaging improvements— which are comparable to those observed in a similar study carried out by Sandison and colleagues [43]— support the conduction of a larger, randomized controlled clinical trial, which is ongoing at six different sites (https://beta.clinicaltrials.gov/study/NCT05894954?cond=Cognitive% 20Decline&term=reversal&rank=5).

## CONSIDERATIONS AND IMPLICATIONS

Identifying and targeting specific putative drivers of cognitive decline with the approach described here represents a fundamental shift in the method by which patients with cognitive decline, or risk for decline, are evaluated and treated. Thus, if further studies prove it to be superior to the current standard of care, such proof would inevitably carry with it far-reaching implications and considerations. One consideration is practicality: the analyses described are more comprehensive than what are currently in use in memory centers, the laboratory data collection more extensive, the behavioral alterations required of the patients more demanding (making health coaches a valuable part of a successful team), the time required by the medical team greater, and the cost significant (although substantially less than an assisted living facility or anti-amyloid antibodies). Further refinement and simplification of the protocol may render it more feasible, accessible, affordable, and ultimately, reimbursable.

Considering the high frequency of failure of clinical trials of pharmaceutical candidates for AD-associated MCI and dementia, which may be in part because they target too few of the network elements driving the pathophysiological process, combining drug candidates with the personalized protocol described here may increase success rate. Furthermore, given the recognized biochemical targets of the interventions, novel pharmaceutical agents may become a key part of an optimal protocol. In addition, new therapeutic targets may emerge from studies of both the inducers of cognitive decline (such as specific pathogens or vascular/endothelial dysfunction) and the mediators (such as specific cytokines, signaling pathways, or trained innate immunity).

In some ways, the multi-pronged therapeutic approach described here is more similar to a surgical protocol than a typical prescription-based medical protocol: teams are important for optimal outcomes, a complex sequence of treatments is required for success, and there is large variability in practitioner success, with some but not all practitioners achieving cognitive improvement in the vast majority of patients. In medical practice, precision medicine might be compared to cardiovascular rehabilitation interventions, which are also comprised of pharmaceutical, nutritional, and exercise protocols. Compassionate use of the protocol in multiple clinics has demonstrated success, but not to the same degree as was observed in the proof-of-concept trial [48].

One important implication is that the availability of a protocol that does not simply retard decline modestly, but actually improves cognition sustainably (some but not all of the first patients, who began treatment in 2012, remain very functional at this time), may provide a powerful stimulus for patients to begin prevention earlier, or undergo treatment during the subjective cognitive impairment (SCI) stage of cognitive decline, and thus it has the potential to reduce dementia rates markedly. One problem with large-scale, public health programs for prevention is that, in the absence of symptoms, patients often feel little incentive to engage in such a program. However, since SCI may last years or even a decade [49], there is opportunity for a protocol that successfully addresses SCI to cut dementia rates sharply. The new availability of blood-based tests such as p-tau 217 should allow more practical early detection, treatment, and monitoring.

Results from precision medicine approaches, as well as others such as the FINGER trial [50], suggest that the common assertion that “There is nothing that will prevent, reverse, or delay the onset of Alzheimer’s disease” is now outdated, and should be retired. It is no longer accurate, and unfortunately it contributes to the nocebo effect [51], creating a false sense of hopelessness and possibly increasing suicide risk [52]. As the public begins to be aware that there are indeed effective approaches available, more people will likely seek prevention or early treatment, and this could have a major impact on the societal burden of dementia. Moreover, the days in which we tell many patients to “come back later” should be terminated— earlier treatment and prevention are clearly superior to treatment during MCI or dementia phases.

However, to accelerate multi-modal, personalized protocols will require fundamental changes in the mechanisms in place for clinical advancement: IRBs, which typically evaluate proposed trials involving single variables such as a drug candidate, are not currently configured for multi-component trial evaluation. A similar situation exists for many funding agencies, in which reviewers are often expert in mono-etiological models and monopharmaceutical approaches rather than in personalized, multi-component therapies.

Although it may be argued that the evaluation of single therapeutics by averaging patients’ responses over unstratified populations allows more scientific rigor and more accurate conclusions, sacrificing patient outcomes for scientific rigor is not a desirable tradeoff. Furthermore, it is likely that AI will allow much more depth in the analysis of multi-variable trials and population-based data, obviating the advantages of single-variable trials.

Reimbursement of multi-component personalized treatments may be challenging, since most healthcare payors lack incentive to reward outcomes (accountable care organizations excepted). However, there are large potential savings for patients, society, and organizations such as long-term care insurers. The total lifetime cost of care for a patient with dementia has been estimated to be over $400,000 [53], with 70% of those costs borne by the family caregivers in the form of unpaid caregiving and out-of-pocket expenses for items ranging from home health support to medications. Effective prevention and early treatment have the potential to reduce that estimate substantially.

In summary, the rationale for utilizing precision medicine protocols for the treatment of patients with cognitive decline, or risk for decline, is based both on the underlying mechanisms driving AD-related signaling and on promising initial results, supporting more extensive studies. The implications for clinical practice, pharmaceutical candidate evaluation, and the societal burden of dementia are profound.
